# Metastatic Lung Adenocarcinoma to the Uterus Mimicking a Primary Uterine Malignancy: A Case Report

**DOI:** 10.1002/cnr2.70706

**Published:** 2026-10-11

**Authors:** Pengpeng Sun, Yue Wang

**Affiliations:** ^1^ Department of Laboratory Medicine Yancheng TCM Hospital Affiliated to Nanjing University of Chinese Medicine Yancheng Jiangsu People's Republic of China; ^2^ Department of Laboratory Medicine Yancheng TCM Hospital Yancheng Jiangsu People's Republic of China; ^3^ Jiangsu Vocational College of Medicine Yancheng Jiangsu People's Republic of China; ^4^ Department of Radiation Oncology Yancheng No. 1 People's Hospital, Affiliated Hospital of Medical School, Nanjing University Yancheng Jiangsu People's Republic of China; ^5^ Department of Radiation Oncology The First People's Hospital of Yancheng Yancheng Jiangsu People's Republic of China

**Keywords:** EGFR mutation, lung adenocarcinoma, misdiagnosis, osimertinib, palliative radiotherapy, uterine metastasis

## Abstract

**Background:**

Lung adenocarcinoma is the most prevalent subtype of non‐small cell lung cancer and exhibits a high tendency for distant metastasis. However, uterine metastasis with synchronous multiorgan involvement is extremely rare and prone to misdiagnosis due to atypical clinical presentations. To date, fewer than 20 cases of lung adenocarcinoma metastasizing to the uterus have been documented globally, most of which present with typical gynecological symptoms, making our atypical case highly educational and complementary to existing clinical data.

**Case:**

Herein, we describe a 77‐year‐old postmenopausal female patient who initially presented with lumbago and dizziness without typical pulmonary or gynecological symptoms, with an ECOG performance status of 1. Imaging studies raised suspicion of a primary uterine malignancy, but pathological biopsy, immunohistochemistry, and genomic testing ultimately confirmed advanced lung adenocarcinoma with multiorgan metastases (uterus, lung, liver, bone). Molecular analysis identified an EGFR exon 21 p.L858R mutation, and the patient received palliative local radiotherapy combined with osimertinib targeted therapy. During treatment, the patient developed mild radiation‐induced fatigue and slight myelosuppression, which were fully relieved after symptomatic supportive care, with no severe unexpected adverse events observed.

**Conclusion:**

This case illustrates the atypical metastatic pattern of lung adenocarcinoma, underscores the pivotal role of pathological and immunohistochemical examinations in differentiating primary from metastatic tumors, and offers a reference for managing diagnostically challenging cases. Early and accurate diagnosis coupled with genomic‐guided personalized therapy is critical for enhancing quality of life and prognosis in patients with advanced lung cancer.

## 1 Introduction

Lung cancer remains the leading cause of cancer‐related mortality globally, and lung adenocarcinoma constitutes approximately 40% of all lung cancer cases [1]. Distant metastasis is a hallmark of advanced lung adenocarcinoma, with typical sites including the brain, bone, liver, and adrenal glands [2]. However, metastasis of lung cancer to the uterus is exceedingly rare. Among secondary tumors of the female genital tract, the ovaries (approximately 76%) and the vagina (approximately 13%) are most frequently involved, whereas the uterus accounts for only about 8% of cases [3, 4]. Because of their nonspecific clinical manifestations and overlapping imaging features, such lesions are easily misdiagnosed as primary uterine tumors.

Primary uterine malignancies, including cervical and endometrial carcinomas, typically manifest with abnormal vaginal bleeding or lower abdominal pain. In contrast, metastatic tumors to the uterus often lack classic gynecologic symptoms, increasing diagnostic difficulty [5]. Moreover, identification of multiorgan lesions may lead to erroneous assignment of the primary tumor origin, resulting in delayed treatment. Thus, for patients with multiorgan lesions of unknown primary origin, integration of pathological biopsy, immunohistochemistry, and genomic profiling is essential to identify the primary site and guide personalized therapeutic strategies.

Uterine metastasis of lung cancer has been documented in fewer than 20 cases in the literature worldwide 3 The present case adds educational value to these reports: (i) the patient presented solely with low back pain and dizziness, without any gynecological symptom; (ii) imaging initially suggested a primary uterine malignancy, which was subsequently refuted by pathological evaluation; and (iii) the metastatic origin was supported by a concordant immunophenotype and by an actionable EGFR mutation detected in the uterine lesion. We report this case to raise awareness of atypical metastatic patterns of lung adenocarcinoma and to reduce the risk of misdiagnosis.

## 2 Case Presentation

A 77‐year‐old postmenopausal woman presented with persistent low back pain for more than 4 months without an identifiable trigger, which she initially ignored. She was subsequently admitted to the Department of Neurology at The First People's Hospital of Yancheng in February 2026 due to dizziness of 1 week's duration and fatigue. The patient had no cough, expectoration, chest tightness, shortness of breath, or other typical pulmonary symptoms, with an ECOG performance status of 1. Cranial computed tomography (CT) revealed no obvious abnormalities. Subsequent positron emission tomography–computed tomography (PET–CT) demonstrated an occupying lesion in the right lower uterine body near the cervix, along with multiple systemic lesions (lungs, liver, bones), which were initially suspicious for a primary uterine malignancy. In February 2026, outpatient curettage was performed; pathological examination showed mucinous tissue in the endocervical canal and chronic inflammation of the cervix. The initial curettage was performed based on abnormal space‐occupying lesions observed on pelvic imaging, without obvious cervical or uterine bleeding symptoms. For further evaluation and management, the patient was admitted to the Department of Radiation Oncology with a working diagnosis of cervical occupying lesion, pulmonary nodules, liver tumor, and bone tumor. Her recent appetite and sleep were satisfactory, bowel and bladder function were normal, and there was no significant weight loss. She had no history of hypertension, diabetes, or coronary heart disease, no family history of malignancy, no history of smoking or alcohol consumption, and was postmenopausal with two prior pregnancies. The patient's detailed clinical diagnostic timeline is summarized in Table 1.

**TABLE 1:** Clinical diagnostic timeline: From initial presentation to follow‐up.

Date	Clinical step	Key findings	Diagnostic significance
Oct 2025 (symptom onset)	Initial presentation	Persistent lower back pain (> 4 months) and dizziness	Non‐specific symptoms; no gynecological complaints
Feb 6, 2026	PET/CT	Uterine mass (SUVmax 12.4); multi‐organ lesions (lung, liver, bone)	Suspected primary uterine malignancy
Feb 10, 2026	Diagnostic curettage	Cervical mucus &chronic inflammation; no malignant cells identified	False negative; diagnostic delay
Feb 25,2026	Endoscopy (gastroscopy + colonoscopy)	Gastric fundus gland polyp (0.4 × 0.4 cm); colonoscopy unremarkable	Ruled out Gl primary
Feb 26, 2026	Pelvic MRI + ultrasound‐guided biopsy	Heterogeneous low‐echo mass at uterine‐cervical junction; core needle biopsy	Targeted tissue sampling
Mar 3, 2026	Histopathology + IHC	Adenocarcinoma confirmed; TTF‐1 (+), NapsinA (+), CK7 (+); PAX8 (−), ER (−), PR (−)	Pulmonary origin confirmed
Mar 2026	Molecular testing	EGFR exon 21 p.L858R mutation detected	TKl sensitivity predicted
Mar 2026	Treatment (MDT decision)	Palliative radiotherapy (30 Gy/10 fractions) + osimertinib (targeted therapy)	Stage IV lung adenocarcinoma
Jul 30, 2026	Follow‐up CT	Uterine tumor and pelvic lymph nodes markedly reduced; pulmonary nodules stable; PR per RECIST 1.1	Partial response

Abbreviations: EGFR, epidermal growth factor receptor; Gy, gray; IHC, immunohistochemistry; MDT, multidisciplinary team; PET/CT, positron emission tomography/computed tomography; PR, partial response; RECIST, response evaluation criteria in solid tumors; SUVmax, maximum standardized uptakeValue; TTF‐1, thyroid transcription Factor‐1.

### 2.1 Auxiliary Examination Results

#### 2.1.1 PET–CT Examination (Early February 2026)

1. An occupying lesion was detected in the right lower uterine body near the cervix, with mild enlargement of bilateral iliac and para‐aortic lymph nodes, multiple diffuse nodules in both lungs, a nodule in liver segment S5, and multiple systemic osteoblastic bone lesions. All lesions showed elevated glucose metabolism (SUVmax ≈12.4), suggestive of malignant disease, likely primary uterine; biopsy of the uterine lesion was recommended for pathological confirmation (Figure 1).
2. Small mediastinal lymph nodes with increased glucose metabolism, requiring close follow‐up to exclude metastasis; arteriosclerosis.
3. Elevated glucose metabolism in the gastric antrum and parts of the colon/rectum, warranting endoscopic evaluation; small hepatic cyst.
4. Age‐related brain changes; bilateral maxillary sinusitis; heterogeneous thyroid density, suggesting ultrasound follow‐up.
5. Degenerative spinal changes.

**FIGURE 1:**
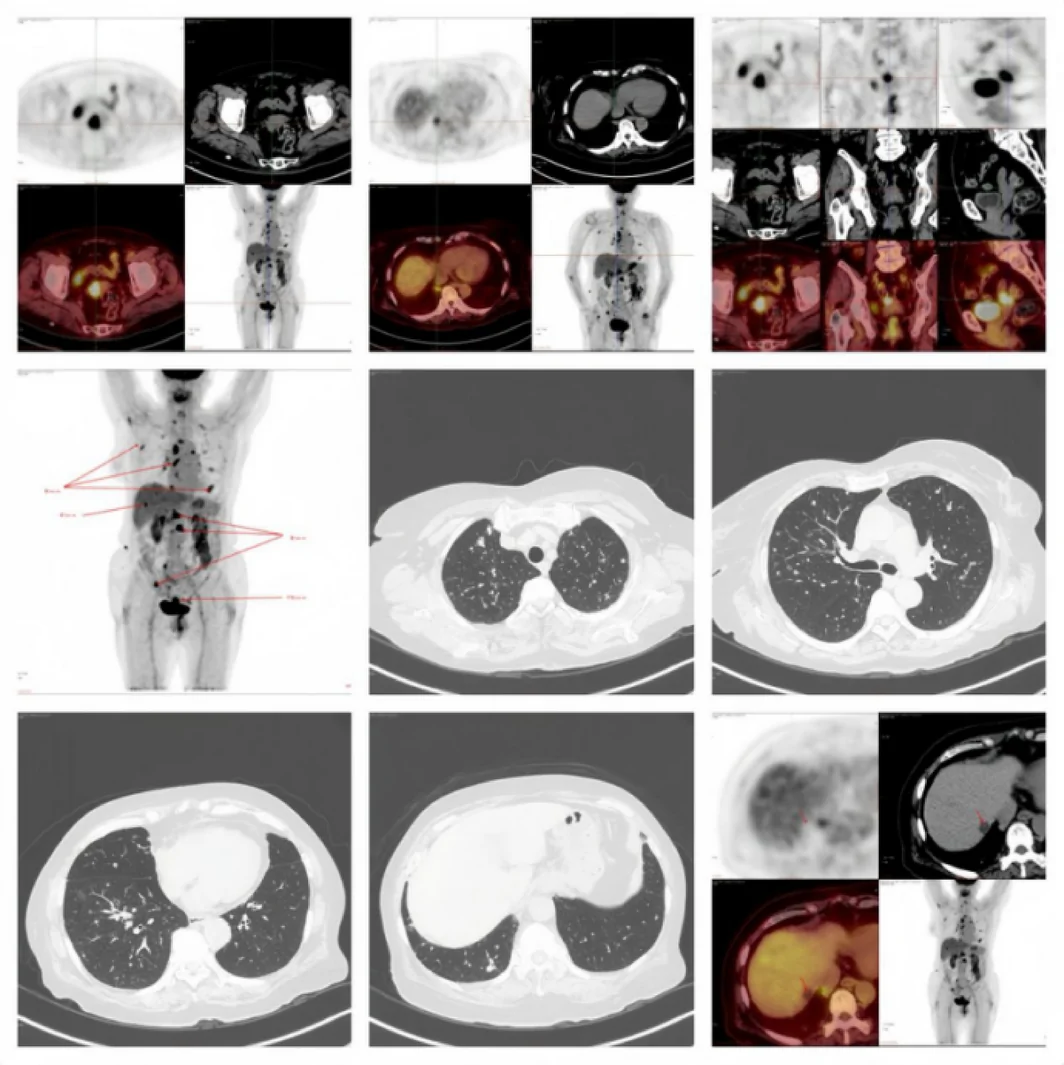
Whole‐body 18F‐FDG PET/CT performed at staging (February 2026). The examination shows a hypermetabolic mass in the right lower uterine segment adjacent to the cervix (arrowhead), multiple bilateral pulmonary nodules, a solitary hepatic lesion in segment V (arrow), enlarged bilateral iliac and para‐aortic lymph nodes, and multiple systemic osteoblastic skeletal lesions, all with increased FDG uptake (maximum standardized uptake value approximately 12.4). Axial and coronal reformations used to delineate the uterine mass and the skeletal lesions are also displayed. Together, these findings initially suggested a primary uterine malignancy with widespread metastases.

#### 2.1.2 Further Inpatient Evaluation

Gastroscopy and colonoscopy (February 25, 2026): A 0.4 × 0.4 cm fundic gland polyp was removed from the gastric fundus; histopathology confirmed a fundic gland polyp. No abnormal findings were noted on colonoscopy.

Pelvic magnetic resonance imaging (MRI) (February 26, 2026): Abnormal signal in the right lower uterine body near the cervix, recommending percutaneous biopsy; abnormal signals in the right ilium and left acetabulum consistent with metastatic disease, suggesting correlation with ECT.

#### 2.1.3 Pathological Examination & Histomorphological Features

Curettage specimens: endocervical canal showed mucinous tissue; cervix showed chronic inflammation with no malignant cells identified.

Uterine percutaneous biopsy specimens showed poorly differentiated adenocarcinoma tissues. Microscopically, tumor cells presented solid nested and sheet‐like growth patterns, with no obvious glandular structural differentiation. Tumor cells had large and irregular nuclei, coarse chromatin, obvious nuclear atypia, increased mitotic figures, and focal stromal invasive growth. The morphological features were highly similar to poorly differentiated lung adenocarcinoma and overlapped partially with primary uterine poorly differentiated adenocarcinoma, resulting in initial morphological diagnostic confusion.

#### 2.1.4 Immunohistochemical and Genomic Findings

Immunohistochemistry: P16 (+), ER (SP1) (0), PR (1E2) (0), Vim (focal +), PAX8 (−), TTF‐1 (8G7G3/1) (+), CDX2 (−), SATB2 (−), CK7 (+), CK20 (−), Villin (−), NapsinA (+). This immunophenotype strongly supports pulmonary tumor origin rather than absolutely excluding primary uterine malignancy, as individual TTF‐1 positivity has been reported in a small number of uterine adenocarcinomas. The combined signature of TTF‐1/Napsin A/CK7 positivity and PAX8/ER/PR negativity, combined with morphological features, is highly suggestive of lung adenocarcinoma metastasis (Figure 2).

**FIGURE 2:**
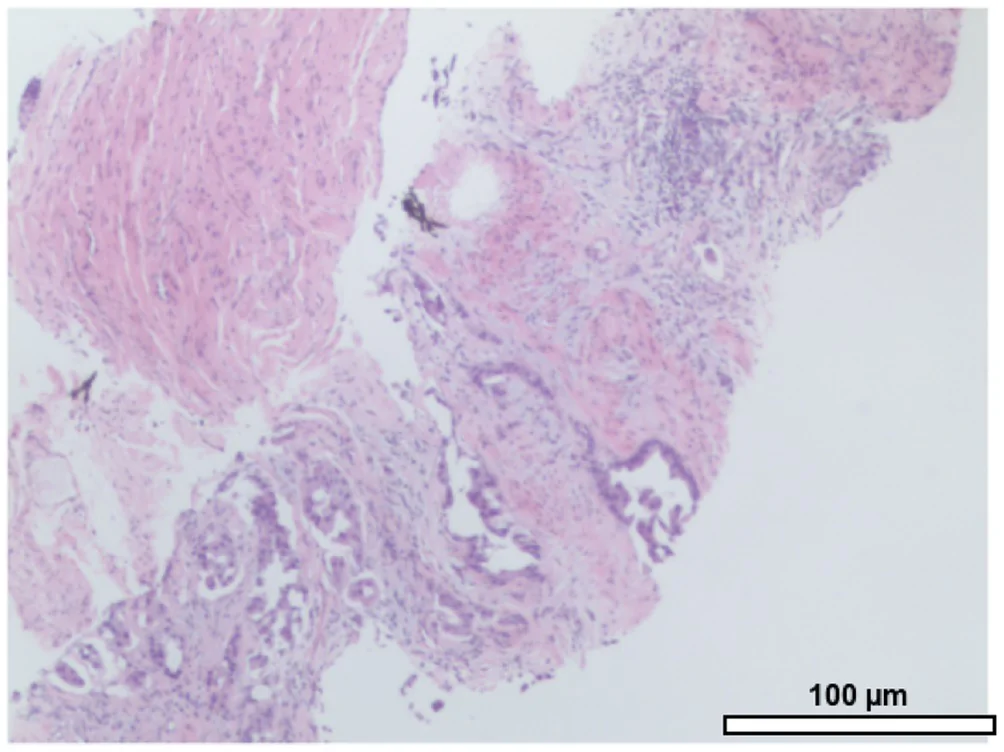
Histology of the ultrasound‐guided percutaneous core‐needle biopsy specimen from the uterine lesion (hematoxylin–eosin staining, original magnification ×10; scale bar = 100 μm). The image shows an infiltrating adenocarcinoma composed of irregular glandular structures with cribriform areas and occasional solid nests, moderate nuclear atypia with prominent nucleoli, and a desmoplastic stroma. No villoglandular, overt endometrioid or endocervical‐type mucinous differentiation was identified. The immunophenotype (TTF‐1, Napsin A and CK7 positive; PAX8, ER and PR negative) was strongly supportive of a pulmonary origin.

Genetic testing: The specimen was derived from uterine cervical‐body junction biopsy tissue, detected by next‐generation sequencing (NGS) full exon panel. EGFR exon 21 p.L858R mutation was definitely detected in uterine lesion tissue, and no other actionable gene alterations were identified, indicating potential sensitivity to tyrosine kinase inhibitor (TKI) therapy.

### 2.2 Diagnosis and Treatment

After multidisciplinary team (MDT) consultation (gynecology, medical oncology, radiation oncology, pathology, radiology, nuclear medicine), the final diagnosis was established as Stage IV advanced lung adenocarcinoma with multiorgan metastases (uterus, lung, liver, bone) and EGFR exon 21 p.L858R mutation.

After exclusion of contraindications, palliative radiotherapy was delivered to systemic osteoblastic metastatic lesions and local uterine mass lesions using 6 MV‐X helical tomotherapy with a planned dose of 30 Gy in 10 fractions. The radiotherapy and osimertinib targeted therapy were administered concurrently, and oral osimertinib was continued routinely during radiotherapy. Strict blood routine and pulmonary function monitoring were performed throughout the treatment to avoid overlapping toxicities such as radiation pneumonitis and myelosuppression. The patient was concurrently prescribed oral osimertinib as targeted therapy.

### 2.3 Follow‐Up

The patient is currently undergoing radiotherapy and targeted therapy. Treatment‐related adverse events, including radiation‐induced fatigue and mild myelosuppression, have improved with supportive care. As of the latest follow‐up on July 30, 2026, contrast‐enhanced CT of the chest and abdomen showed a marked reduction in the uterine tumor and pelvic lymph nodes, with pulmonary nodules essentially unchanged; the response was classified as partial response (PR) per RECIST 1.1. Long‐term follow‐up will be conducted to monitor disease progression and quality of life.

## 3 Discussion

This case illustrates the diagnostic challenges of advanced lung adenocarcinoma with multiorgan metastases initially misdiagnosed as a primary uterine tumor. Unlike previously reported cases that mostly presented with abnormal vaginal bleeding, this patient had only atypical low back pain and dizziness without any classic gynecological or pulmonary symptoms, which is extremely rare in clinical practice. Key diagnostic and therapeutic considerations are discussed below.

### 3.1 Causes of Misdiagnosis and Diagnostic Reasoning Evolution

The primary reasons for misdiagnosis include the extreme rarity of uterine metastasis from lung adenocarcinoma and the absence of specific clinical manifestations. The patient's lack of classic gynecological bleeding symptoms reduced clinicians' vigilance for uterine primary tumors; however, PET‐CT showed a dominant high‐metabolism uterine space‐occupying lesion combined with widespread systemic multi‐organ metastases, which conformed to the metastatic characteristics of advanced uterine malignant tumors, thus leading to the initial judgment of primary uterine malignancy. Furthermore, initial curettage only detected chronic cervical inflammation and mucinous tissue without malignant cells, contributing to diagnostic delay. This is attributable to the limited sampling area of curettage and potential heterogeneous distribution of metastatic foci in the uterus. With the completion of uterine percutaneous biopsy, IHC detection and molecular genetic testing, the tumor primary site was finally corrected to the lung, completing the differential diagnosis evolution from suspected uterine primary to confirmed lung metastasis.

### 3.2 Pathological and Immunohistochemical Differential Diagnostic Value

The uterine metastatic lesion showed poorly differentiated adenocarcinoma morphology with solid growth and no obvious glandular differentiation, which overlaps with the morphological characteristics of primary uterine poorly differentiated adenocarcinoma, leading to morphological diagnostic difficulties. Immunohistochemistry is indispensable for distinguishing primary from metastatic tumors. In this case, positive staining for TTF‐1 and NapsinA (specific markers of lung adenocarcinoma) and negative staining for PAX8, ER, and PR (typical markers of primary uterine adenocarcinoma) strongly support the pulmonary origin of the tumor, combined with clinical and imaging evidence [6, 7]. It is worth noting that TTF‐1 has low positive expression in a small number of endometrial adenocarcinomas, so a single IHC marker cannot be used for absolute diagnosis. The combined multi‐index immunohistochemical panel achieves high diagnostic accuracy.

### 3.3 Therapeutic Strategy and Safety Analysis

The therapeutic goal in metastatic lung adenocarcinoma is symptom relief, disease control, and quality‐of‐life improvement. Palliative radiotherapy effectively alleviates bone pain and local mass effect from uterine metastasis, enhancing patient functional status [8]. Targeted therapy is the first‐line standard for advanced lung adenocarcinoma harboring actionable mutations. The *EGFR* exon 21 p.L858R mutation detected herein is a common sensitizing alteration in lung adenocarcinoma, predicting favorable response to TKIs such as osimertinib [9]. Osimertinib selectively inhibits mutant EGFR, suppressing tumor proliferation and metastasis with superior efficacy and milder toxicity compared with conventional chemotherapy [10]. Concurrent radiotherapy and targeted therapy have potential overlapping toxicities, especially radiation pneumonitis, so individualized dose adjustment and strict clinical monitoring are required. In this case, the combined regimen was well‐tolerated with only mild controllable adverse events. This case confirms the clinical feasibility and safety of this combined regimen, while its long‐term efficacy needs further long‐term follow‐up verification.

### 3.4 Comparison With Previously Reported Cases

Uterine metastasis from lung cancer remains an extremely rare event, with fewer than 20 cases reported in the English‐language literature, mostly as isolated case reports [3]. Yu et al. recently described the largest retrospective series to date (five patients, age range 33–66 years); the predominant presentations were abnormal vaginal bleeding (3/5) or abdominal pain and bloating (1/5), one case was asymptomatic and discovered incidentally, and the metastatic sites included the uterine corpus (three cases), a cervical leiomyoma (one case) and the cervical endometrium (one case) [3]. Compared with these reports, the present case has several distinctive features: (i) it occurred in an older patient (77 years); (ii) the presenting symptoms (low back pain and dizziness) were entirely non‐gynecological; (iii) imaging initially suggested a primary uterine malignancy; (iv) a negative initial D&C caused diagnostic delay; and (v) the patient received a combination of palliative radiotherapy with osimertinib, a management sequence that has rarely been reported in uterine‐metastasis cases. Metastases of lung cancer to the female genital tract most frequently involve the ovaries, whereas uterine involvement accounts for only a small fraction [4], which may partly explain why uterine metastases are so seldom encountered.

### 3.5 Clinical Implications

This case underscores that clinicians should not rely solely on initial imaging when evaluating patients with multiorgan lesions of unknown origin, especially those with atypical symptoms. Comprehensive integration of clinical data, pathological biopsy, immunohistochemistry, and genomic testing is mandatory to identify the primary tumor. Heightened awareness of rare metastatic sites such as uterine involvement from lung adenocarcinoma is essential to prevent misdiagnosis and delayed treatment. Furthermore, genomic‐guided personalized therapy represents the future of advanced lung cancer management. Early precise diagnosis and individualized intervention can substantially improve patient quality of life and prognosis, providing valuable lessons for similar clinical scenarios.

## 4 Conclusion

In conclusion, uterine metastasis from lung adenocarcinoma is an extremely rare clinical manifestation with atypical symptoms and overlapping imaging and pathological features, which is highly prone to misdiagnosis as primary uterine malignancy. This case is unique in that the elderly postmenopausal patient had no classic gynecological or pulmonary symptoms, with multi‐organ synchronous metastasis as the initial presentation. Pathological biopsy combined with multi‐index immunohistochemistry and molecular genetic testing is essential for accurate identification of tumor primary origin. For advanced EGFR‐mutant lung adenocarcinoma with rare uterine metastasis, the combined treatment of palliative radiotherapy and osimertinib is clinically feasible and well‐tolerated, which can effectively relieve patient symptoms and improve quality of life. Clinicians should strengthen the recognition of atypical metastatic patterns of lung cancer and conduct a comprehensive multi‐dimensional evaluation to achieve early diagnosis and precise individualized treatment.

## Author Contributions

**Pengpeng Sun:** conceptualization, validation, resources, data curation, writing – review and editing, visualization, supervision, project administration, funding acquisition. **Yue Wang:** formal analysis, investigation, methodology, software, writing – original draft.

## Funding

The authors declare that financial support was received for the research and publication of this article. This work was supported by the Fund of the Jiangsu Vocational College of Medicine University‐Local Collaborative Innovation Research Project (program no. 202491717).

## Ethics Statement

This study was approved by the Ethics Committees of The First People's Hospital of Yancheng. Written informed consent for publication of clinical data and imaging findings was obtained from the patient and her family. All procedures were performed in accordance with the Declaration of Helsinki.

## Conflicts of Interest

The authors declare no conflicts of interest.

## Data Availability

The data that support the findings of this study are available from the corresponding author upon reasonable request.
